# Integrative Analysis of Differential lncRNA/mRNA Expression Profiling in *Helicobacter pylori* Infection-Associated Gastric Carcinogenesis

**DOI:** 10.3389/fmicb.2020.00880

**Published:** 2020-05-08

**Authors:** Nianshuang Li, Yaobin Ouyang, Sihai Chen, Chao Peng, Cong He, Junbo Hong, Xiaoyu Yang, Yin Zhu, Nong-Hua Lu

**Affiliations:** ^1^Department of Gastroenterology, The First Affiliated Hospital of Nanchang University, Nanchang, China; ^2^Institute of Digestive Disease, The First Affiliated Hospital of Nanchang University, Nanchang, China

**Keywords:** *Helicobacter pylori*, RNA sequencing, lncRNA, mRNA, gastric cancer

## Abstract

*Helicobacter pylori* (*H. pylori*) infection is the greatest known risk factor for gastric cancer (GC). Long non-coding RNAs (lncRNAs) are implicated in multiple biological processes. However, their contribution in *H. pylori*-associated GC remains largely unknown. We performed transcriptome sequencing to investigate differential lncRNA and mRNA expression profiles in gastric AGS cells infected with the *H. pylori* strain 7.13 or 43504. We identified significantly differentially expressed (SDE) mRNAs and lncRNAs following *H. pylori* infection. A co-expression network of lncRNAs and mRNAs was constructed via WGCNA analysis. Moreover, several of the most significantly upregulated genes were selected for further validation by qRT-PCR analysis in *H. pylori*-infected gastric cells and transgenic INS-GAS mice. We finally evaluated these genes in human GC tissues. A total of 158442 genes were identified between uninfected and infected cells. Of these, 298 mRNAs and 73 lncRNAs were consistently differentially expressed following infection with the *H. pylori* 7.13 and 43504 strains, respectively. The expression levels of most upregulated mRNAs (DDIT4, NDRG1, CHAC1, IL32, RELB, CTH, and SLC7A1) and lncRNAs (lncRNA36068, lncRNA51663, lncRNA49853, lncRNA49852, and FLJ46906) were validated by qRT-PCR analysis. We found that *H. pylori* infection significantly induced the transcript levels of the coding genes RELB and SLC7A11 in *in vitro* and *in vivo* assays, which was supported by their high expression levels in GC tissues. In addition, lncRNA51663 and FLJ46906 were remarkably increased in *H. pylori*-infected cells and consistently overexpressed in human GC tissues compared to adjacent normal tissues. Our study identified mRNA and lncRNA expression profiles related to *H. pylori* infection. These results may provide important insights regarding lncRNAs in *H. pylori*-induced gastric carcinogenesis.

## Introduction

Although it is steadily declining in incidence, gastric cancer (GC) is still the third leading cause of cancer deaths worldwide. An infection with the gram-negative microaerophilic bacterium *Helicobacter pylori* (*H. pylori*) has been identified as a group I carcinogen by the IARC/WHO. *H. pylori* colonizes the gastric mucosa of approximately half of the world’s population, mainly in East Asian countries (Kusters et al., 2006; Amieva and Peek, 2016). In general, most individuals infected with *H. pylori* are asymptomatic. However, only 10–15% of individuals infected with *H. pylori* develop duodenal ulcer diseases, and 1–3% of individuals are estimated to develop GC (Li et al., 2018). We have previously shown that the release of ROS, the DNA damage response, and the inflammatory response are increased in gastric epithelial cells infected with *H. pylori* (Xie et al., 2018; Hu et al., 2019). Many randomized controlled trials have indicated that eradication of *H. pylori* can significantly reduce the risk of GC (Li et al., 2014; Li et al., 2019). Although different pathogenic mechanisms of gastric carcinogenesis have been identified in multiple studies, the underlying molecular mechanism remains unknown.

Long coding RNA (lncRNA) is a heterogeneous class of transcripts longer than 200 nucleotides that do not have protein-coding capacity. Many lncRNAs share similar features with mRNAs, including transcription with RNA polymerase II, a 5′ 7-methylguanosine cap and a 3′ poly(A) tail (Kopp and Mendell, 2018). Despite not being translated into proteins, lncRNAs regulate gene expression at various levels through diverse mechanisms. Many of them have been reported to mediate protein localization, interact with chromatin modification complexes, or directly affect transcriptional levels. Furthermore, some lncRNAs have been involved in posttranscriptional processes, including alternative splicing, mRNA decay, protein translation and protein stability (Wang and Chang, 2011; Marchese et al., 2017). The application of next-generation sequencing to a growing number of transcriptomes has revealed that deregulation of lncRNAs is related to different human diseases, including cancer and cardiovascular diseases (Bhan et al., 2017; Sallam et al., 2018). Several common lncRNAs have been determined to modulate cellular biological processes in cancer development, such as cell survival, inflammatory response, cell migration, and stem cell characteristics. HOX Transcript Antisense RNA (HOTAIR), one of the most widely studied lncRNAs, is highly expressed in a variety of solid malignancies and is correlated with metastasis and poor prognosis (Tang and Hann, 2018). Furthermore, the upregulation of HOTAIR has been reported to promote cisplatin resistance in lung adenocarcinoma cells via the regulation of the cyclin-dependent kinase inhibitor p21 (Liu et al., 2013). Another well-characterized lncRNA, metastasis-associated lung adenocarcinoma transcript-1 (MALAT1), has also been investigated as a potential adverse prognostic marker in the progression of many types of cancer (Amodio et al., 2018).

Recent studies have revealed the dysregulation of lncRNAs in gastric tumorigenesis and progression. Wei et al. indicated that lncRNA gastric cancer metastasis-associated long non-coding RNA (GMAN) was significantly overexpressed in gastric tumor tissues. High levels of GMAN promoted gastric tumor metastasis and adverse survival through the regulation of Ephrin A1 translation (Zhuo et al., 2019). Additionally, increased expression of gastric cancer-associated lncRNA (GClnc1) contributed to gastric carcinogenesis and cancer development (Sun et al., 2016). *H. pylori* infection is the strongest known risk factor for gastric cancer. However, the role of lncRNAs in the pathogenesis of *H. pylori* infection remains largely unknown.

In this study, we carried out whole-transcriptome sequencing in gastric cancer AGS cells infected with the CagA^+^ *H. pylori* strain 43503 or 7.13. We identified significantly differentially expressed (SDE) mRNAs and lncRNAs following *H. pylori* infection and revealed a potential relationship between the SDE mRNAs and lncRNAs. Furthermore, we validated the expression of several novel SDE genes in human GC tissues, *H. pylori*-infected cells and animal models.

## Materials and Methods

### *H. pylori* Strains and Cell Culture

The CagA^+^VacA^+^ *H. pylori* ATCC43504 (NCTC11637), 7.13 and PMSS1 strains were used in this study. *H. pylori* bacteria were cultured on tryptic soy agar plates (BD, Biosciences, Cockeysville, MD, United States) with 5% sheep blood. Human gastric epithelial AGS cells were cultured in DMEM/F12 (Gibco, Life Technologies, Carlsbad, CA, United States) supplemented with 10% foetal bovine serum (FBS) and 1% penicillin/streptomycin (Gibco). To perform infection experiments, *H. pylori* strains were then cultured in Brucella broth (BD Bioscience) supplemented with 10% FBS overnight at 37°C under microaerobic conditions. The OD_600 nm_ of bacterial suspensions was detected. Just prior to infection, AGS cells were transferred to DMEM/F12 medium containing 10% FBS. AGS cells were cocultured with the *H. pylori* ATCC43504, 7.13 or PMSS1 strain at a multiplicity of infection (MOI) of 100 for 6 h.

### INS-GAS Mice Were Infected With *H. pylori*

All procedures carried out on animals were approved by the Ethics Committee of The First Affiliated Hospital of Nanchang University. Transgenic insulin-gastrin (INS-GAS) mice on the FVB background, aged 5–8 weeks, were challenged with 2 × 10^8^ colony forming units (CFU)/ml bacterial suspension of *H. pylori* PMSS1 once every other day for a total of five times. After 4 months with *H. pylori* infection, mice were euthanized, and gastric tissues were harvested for histologic examination and qRT-PCR analyses.

### RNA Isolation and Whole-Transcriptome Sequencing

After infection with *H. pylori*, cells were washed at least four times with PBS to remove bacteria. Total RNA was isolated from gastric specimens and gastric cells using TRIZOL reagent, according to the manufacturer’s instructions (Invitrogen, Life Technologies, Carlsbad, CA, United States). For RNA-sequencing (RNA-seq), RNA quantity was evaluated with a NanoDrop 2000 (Thermo Fisher Scientific), and RNA integrity was assessed with an Agilent 5200 Fragment Analyzer (Agilent Technologies). Then, rRNAs were removed from the total RNA and fragmented for cDNA synthesis (Tiangen biotech, Beijing, China). The double-stranded cDNA products were amplified and purified to produce the final cDNA library. Finally, all libraries were sequenced by BGISEQ-500 sequencing platform.

### mRNA and lncRNA Expression Profiling

After sequencing, RNA reads of each sample were filtered with SOAPnuke software, which was developed by the Beijing Genome Institute (Cock et al., 2010), to remove low-quality reads that contain adaptor contamination, more than 20% low-quality bases (base quality <10) and 5% undefined nucleotides [N] on the reads. The remaining clean reads were aligned to the reference genome using HISAT (Kim et al., 2015). For mRNA profiling, clean reads were mapped to the human reference genome. For lncRNAs, the clean reads were mapped to the non-coding RNA transcriptome. The human genome version NCBI GCF_000001405.38_GRCh38.p12 were used. Then, all the mRNA and lncRNA expression profile were analyzed. RSEM was performed to estimate and quantify gene expression (Li and Dewey, 2011). The method of fragments per kilobase of exon per million reads mapped (FPKM) was employed to normalize gene expression. Additionally, DESeq2 package was applied to identify the differential expressed genes (Love et al., 2014). When the original *Q*-value (adjusted *P*-value) is less than 1e-5, the presented *Q*-value was defined as 0. The web-based tool Morpheus^1^ was conducted for hierarchical clustering and visualization of heatmap. Functional annotation enrichment analyses for gene ontology (GO) and Kyoto Encyclopedia of Genes and Genomes (KEGG) pathway were performed. The phyper function of the R software was used for KEGG enrichment analysis. The *Q*-value is the adjusted *P*-value, taking into account of computing False discovery rate (FDR).

### The Cancer Genome Atlas Database analysis

To analyze gene expression in human GC tissues, the expression data of SDE mRNAs, including DDIT4 (DNA-damage-inducible transcript 4), NDRG1 (N-myc downstream regulated gene 1), CHAC1 (ChaC glutathione specific gamma-glutamylcyclotransferase 1), IL-32 (Interleukin 32), RELB (RELB Proto-Oncogene, NF-KB Subunit), CTH (cystathionine gamma-lyase) and SLC7A11 (solute carrier family 7 member 11) from 30 normal and 343 stomach adenocarcinoma (STAD) samples were obtained from TCGA database.

### Tissues, Validation of the SDE mRNAs and lncRNAs

A total of 25 human gastric adenocarcinoma and corresponding adjacent normal tissues were obtained from surgical samples without adjuvant therapy. All specimens were provided by The First Affiliated Hospital of Nanchang University. The study protocol was approved by the Ethics Committee of The First Affiliated Hospital of Nanchang University.

The expression of SDE mRNAs (DDIT4, NDRG1, CHAC1, IL-32, RELB, SLC7A11, CTH) and lncRNAs (lncRNA36068, lncRNA51663, lncRNA49853, lncRNA49852, FLJ46906) was assayed using qRT-PCR as previously described (Buti et al., 2011). These lncRNA transcripts can be localized to specific genomic locations as follows: lncRNA36068 (NC_000001.11:633535-634924), lncRNA51663 (NC_000019.10:47241441-47243250), lncRNA49853 (NC_000016.10:29262273-29264479), lncRNA49852 (NC_000016.10:29256044-29257624), and FLJ46906 (NC_000006.12:138691611-138697629). The primers were designed as shown in Supplementary Table S1. Briefly, total RNA was extracted using TRIZOL reagent. Then, qPCR was performed in the QuantStudio 5 Real-time PCR system (Life Technologies) using the SYBR Fast Master Mix (Thermo Fisher Scientific). Relative transcript levels were normalized to endogenous GAPDH.

### Co-expression Network Analysis

The weighted gene co-expression network analysis (WGCNA) R package was used to analyze the co-expression network between the SDE mRNAs and lncRNAs.

### Statistical Analysis

All statistical analyses were performed using GraphPad Prism 8.0 Software and SPSS 20.0 software. The distribution of characteristics between two groups of continuous variables were determined by Student’s *t*-test. *P* < 0.05 was considered to indicate a statistically significant difference (^∗∗∗^*p* < 0.001, ^∗∗^*p* < 0.01, ^∗^*p* < 0.05).

## Results

### Sequencing Results and Quality Control in Gastric Epithelial Cells Infected With *H. pylori*

**FIGURE 1:**
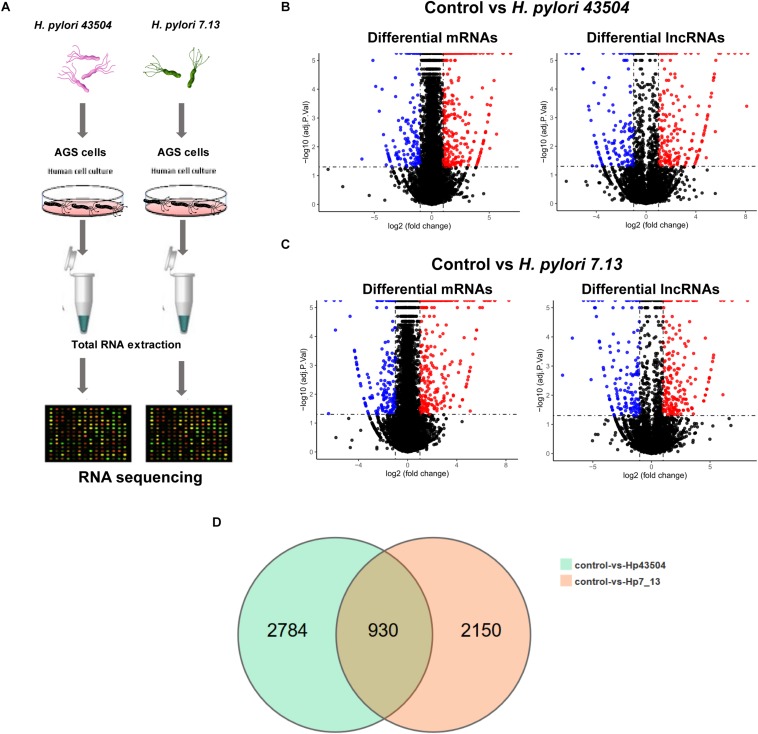
Overview of the RNA sequencing results in gastric epithelial cells infected with *H. pylori*. **(A)** Schematic view of the experimental workflow for transcriptome sequencing in AGS cells infected with *H. pylori*. **(B)** The volcano plot shows the differentially expressed mRNAs and lncRNAs between uninfected and *H. pylori* 43504-infected cells. **(C)** The volcano plot shows the differentially expressed mRNAs and lncRNAs between uninfected and *H. pylori* 7.13-infected cells. **(D)** The Venn diagram shows overlapping genes of significantly differentially expressed genes following *H. pylori* infection.

To identify mRNA and lncRNA expression profiles correlated with *H. pylori* infection, AGS gastric epithelial cells were cocultured with *H. pylori* 43504 or 7.13. The basic workflow is shown in Figure 1A. By performing RNA-seq, a total of 1020.49 million raw reads were generated from 9 RNA samples (3 independent samples from each group). After filtering the reads, 983.45 million clean reads were obtained.

Due to *H. pylori* infection of cells, 52.75–90.7% of the clean reads were aligned to the human reference genome. As a result, more than 99% of all RNA transcripts were mRNAs and lncRNAs (Supplementary Figure S1A). The Pearson’s correlation coefficient that reveals all the gene variation between samples, showed a correlation value of *r* > 0.9 (Supplementary Figure S1B). Principle component analysis (PCA) showed differences in gene expression between the control groups and *H. pylori*-infected groups. However, the replicate samples of each group did not seem to cluster together (Supplementary Figure S1C). There were two possible explanation for this discrepancy. Firstly, different cell passages were performed for replicated samples in each group. Secondly, gastric epithelial cells were exposed to live *H. pylori* bacteria that may lead to the variation among replicates.

### Identification of Significantly Differentially Expressed mRNAs in *H. pylori*-Infected Gastric Cells

**FIGURE 2:**
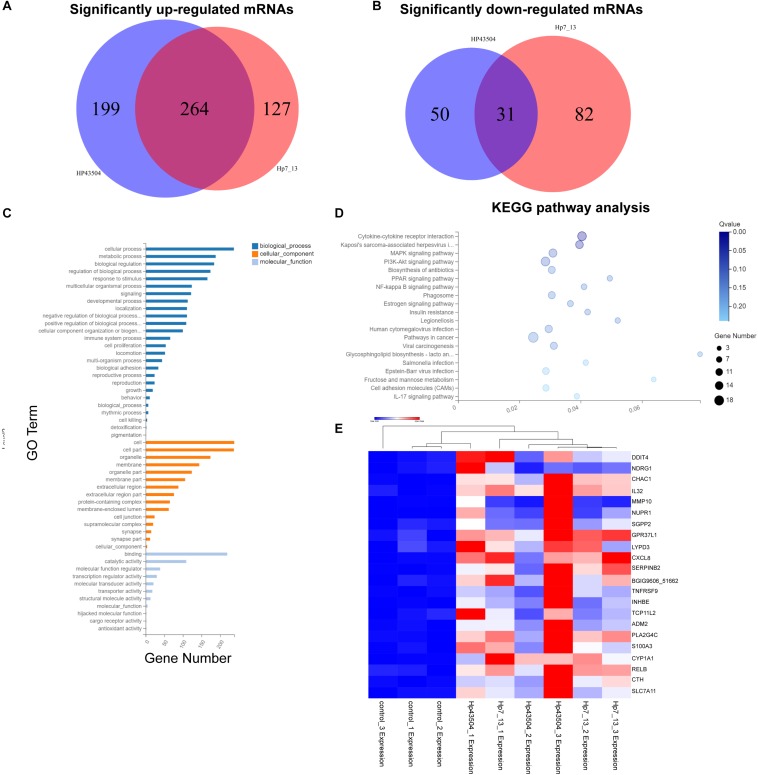
Differential mRNA expression profiling in gastric cells infected with *H. pylori*. **(A,B)** Venn diagrams of the significantly upregulated **(A)** or downregulated **(B)** mRNAs with [log2(FC)] ≥ 1 and *Q*-value ≤ 0.001. **(C)** GO annotation analysis for the consistent SDE genes. The three classes of GO terms, including biological process, cellular component and molecular function, are represented in different colors. **(D)** KEGG pathway enrichment analysis for the consistent SDE genes. The Rich Ratio of *x*-axis refers to the ratio of selected gene numbers annotated in this pathway terms to all gene numbers annotated in this pathway term. The calculating formula is Rich Ratio = Term Candidate Gene Number/Term Gene Number. The size and color of the bubbles represent the number of SDE genes enriched in the pathway and enrichment significance, respectively. The *Q*-value is the adjusted *P*-value. **(E)** The heatmap shows the most significantly upregulated mRNAs in both *H. pylori* 7.13- and 43504-infected cells.

A total of 26696 mRNAs, 17627 lncRNAs and 114119 circRNAs have been identified following *H. pylori* 43504 or *H. pylori* 7.13 infection in AGS cells. In detail, 25037 mRNAs and 11802 lncRNAs were identified following *H. pylori* 43504 infection (Figure 1B). A total of 24781 mRNAs and 11109 lncRNAs were identified following *H. pylori* 7.13 infection (Figure 1C). What’s more, the criteria of [log2(FC)] ≥ 1 (log2 (fold change) and *Q*-value ≤ 0.001, was used to select the significantly differential expression of genes. A total of 930 genes (298 mRNAs, 74 lncRNAs and 558 circRNAs) were significantly differentially expressed in both *H. pylori* 43504- and 7.13-infected cells (Figure 1D). A heatmap was generated to show the expression profiles of SDE genes (Supplementary Figure S1C). We found that 298 mRNAs of these genes were altered following *H. pylori* infection (Supplementary Table S2). In detail, we identified 479 SDE mRNAs between the control and *H. pylori* 43504 groups and 443 mRNAs between the control and *H. pylori* 7.13 groups (Supplementary Tables S3, S4). Specifically, we found that both *H. pylori* 43504 and 7.13 induced the upregulation of 264 mRNAs and the downregulation of 31 mRNAs (Figures 2A,B).

A GO annotation analysis was used to identify the function of these SDE mRNAs. As a result, cellular process was mostly annotated in the biological process category. With regard to the cellular component category, the most highly abundant GO terms were cell and cell part. Regarding to molecular function, the top three GO terms were binding, catalytic activity and molecular function regulation (Figure 2C). Moreover, KEGG pathway enrichment analysis was performed for each group. The SDE genes were also significantly enriched in 20 signaling pathways. Specifically, the results showed that the significantly enriched KEGG pathways were cytokine-cytokine receptor interaction (*Q*-value = 0.01) and Kaposi’s sarcoma-associated herpesvirus infection (*Q*-value = 0.04). Fifteen SDE genes such as IL32, IL33, CXCR2, CXCL8, IL23A and so on were linked to the pathway of cytokine-cytokine receptor interaction. (Figure 2D). These results suggest that the signaling pathways associated with cellular proliferation and inflammation might be involved into the pathogenesis of *H. pylori* infection. The most significantly differentially expressed mRNAs were determined, and 22 mRNAs were most significantly upregulated in both *H. pylori* 43504- and 7.13-infected cells based on the criteria of [log2(FC)] ≥ 2 (log2-fold change), basal expression of mRNAs in *H. pylori*-infected groups ≥ 1 with *Q*-value ≤ 0.001 (Figure 2E and Supplementary Table S5).

### Validation of the Significantly Differentially Expressed Genes Through *in vitro* and *in vivo* Studies

**FIGURE 3:**
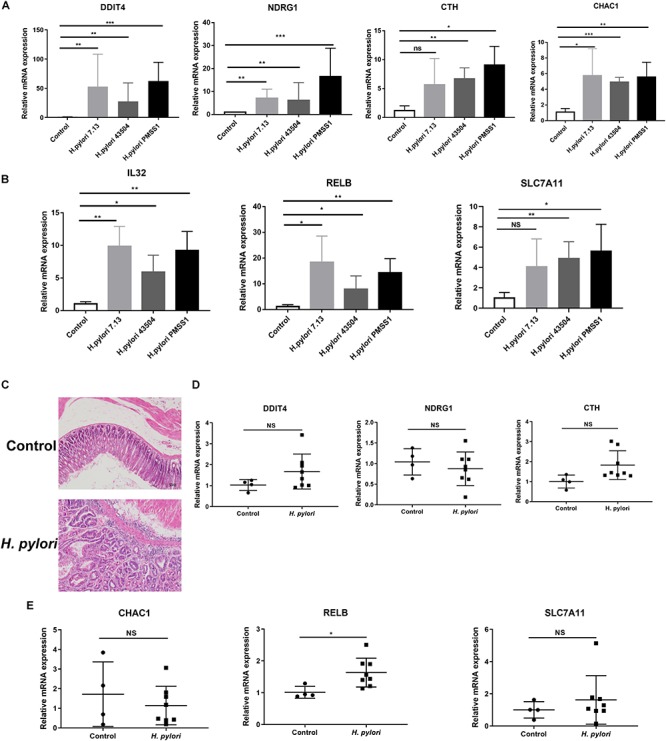
Validation of the significantly differentially expressed genes in *H. pylori*-infected cells and INS-GAS mice. **(A,B)** Gastric epithelial AGS cells were cocultured with CagA^+^ *H. pylori* 43504, 7.13 and PMSS1 strains for 6 h. A qRT-PCR analysis was performed to assess the transcript levels of DDIT4, NDRG1, CTH, CHAC1, IL32, RELB, and SLC7A11. **(C)** H&E staining was performed for the gastric histopathology of INS-GAS mice at 4 months post-infection with the *H. pylori* PMSS1 strain. The representative images are shown for the histopathology of uninfected and infected mice. **(D,E)** The qRT-PCR analysis was performed to assess the SDE mRNAs levels in *H. pylori*-infected INS-GAS mice. The expression levels of DDIT4, NDRG1, CTH, CHAC1, RELB, and SLC7A11 were shown. The relative quantification used GAPDH as an internal control. *p < 0.05; **p < 0.01; ***p < 0.001 (vs. control).

To investigate the potential genes involved in *H. pylori* infection-associated gastric carcinogenesis, we validated the SDE mRNAs expression of DDIT4, NDRG1, CHAC1, IL32, RELB, CTH, and SLC7A1 in cells and animal models infected with *H. pylori*. Because these genes showed higher basic expression and fold changes, as shown in Supplementary Tables S5, S7. Due to the highly variable pathogenicity of *H. pylori* strains, gastric epithelial cells were cocultured with the CagA^+^ *H. pylori* 43504, 7.13 and PMSS1 strains for 6h. The data showed that *H. pylori* infection strongly induced the transcript levels of these coding genes (Figures 3A,B). To further support these *in vitro* data, we used the hypergastrinemic INS-GAS mouse model to develop *H. pylori* infection-associated gastric diseases. Transgenic INS-GAS mice have been reported to develop dysplasia and carcinoma with *H. pylori* infection (Lofgren et al., 2011). INS-GAS mice were challenged with Brucella broth as the negative control or with *H. pylori* strain PMSS1 for 4 months. The gastric mucosal histopathology showed that chronic *H. pylori* infection induced significantly severe inflammation and epithelial hyperplasia (Figure 3C). Because mice do not produce IL-32 (Nakayama et al., 2012), we performed qRT-PCR analysis to assess the levels of DDIT4, NDRG1, CTH, CHAC1, RELB, and SLC7A11 in gastric tissues. We found that DDIT4, CTH, RELB, and SLC7A11, but not NDRG1 and CHAC1, increased in gastric tissues at 4 months post-infection (MPI). However, only RELB was shown to be statistically significantly upregulated after *H. pylori* infection (Figures 3D,E). RELB as a member of the NF-κB family plays an important role in host inflammation. Our data indicated that RELB may be essential for the *H. pylori*-induced host inflammatory response. Taken together, these findings suggested that some genes, including RELB, DDIT4, CTH and SLC7A11, might be implicated in the *H. pylori* infection pathogenesis.

### Expression of SDE Genes in Human GC Tissues

**FIGURE 4:**
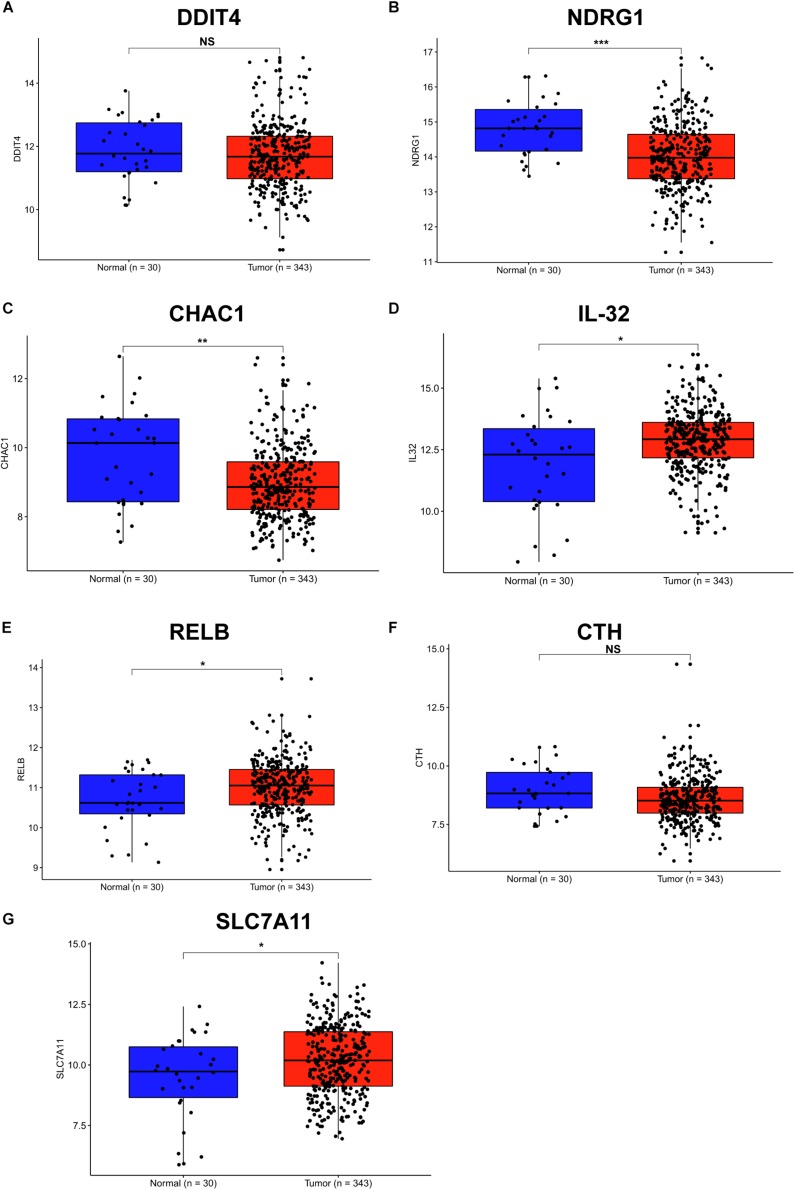
Expression of SDE genes between human GC and adjacent normal tissues from the TCGA database. **(A–G)** Expression of DDIT4 **(A)**, NDRG1 **(B)**, CHAC1 **(C)**, IL-32 **(D)**, RELB **(E)**, CTH **(F)**, and SLC7A11 **(G)** in GC and normal gastric tissues from the TCGA database are shown. Expression values of genes are log2-transformed. A *P*-value of <0.05 was considered statistically significant.

*Helicobacter pylori* infection is the strongest recognized risk factor for GC. Therefore, we aimed to explore the expression of these SDE genes in human GC tissues according to the TCGA database, and 343 GC and 30 normal tissues were used for this study. Consistent with the results in *H. pylori*-infected cells and INS-GAS mice, we found that the gene levels of IL-32, RELB and SLC7A11 were significantly increased in stomach adenocarcinoma tissues compared with those in normal tissues (Figures 4D,E,G). Conversely, DDIT4, NDRG1, and CHAC1 showed lower levels in stomach tumor tissues than that in normal tissues (Figures 4A–C). There were no significant differences in the CTH expression between GC and normal tissues (Figure 4F). These findings further indicated that RELB, SLC7A11, and IL-32 might contribute to *H. pylori* infection-induced gastric carcinogenesis.

### Overview of the lncRNA Expression Profile and the Significantly Expressed lncRNAs in *H. pylori*-Infected Gastric Cells

**FIGURE 5:**
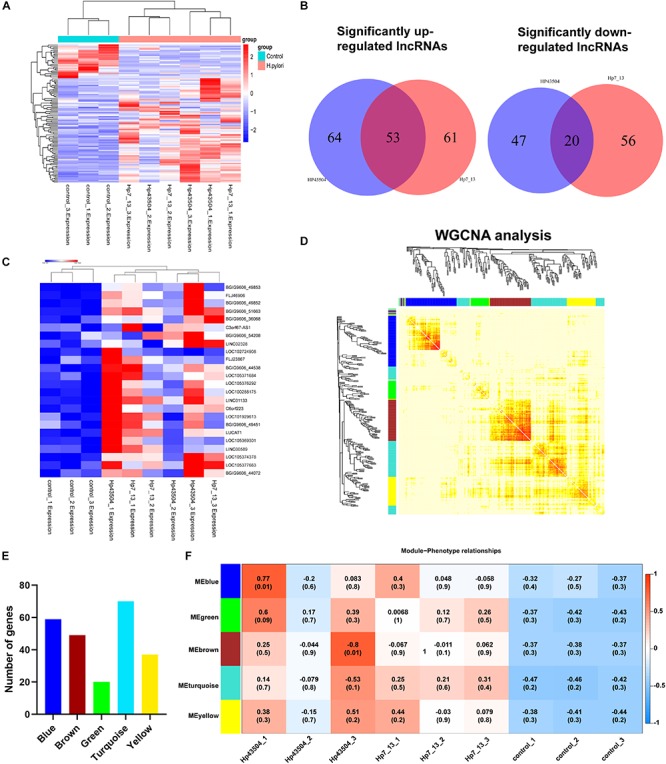
Integrative analysis of differential lncRNAs expression profiling and construction of the lncRNA-mRNA co-expression network in gastric cells infected with *H. pylori*. **(A)** The heatmap showed expression levels of differential lncRNAs with the criteria of | log2(FC) | ≥ 1, *Q*-value ≤ 0.001. **(B)** Venn diagrams of the significantly up- or downregulated lncRNAs following *H. pylori* infection. **(C)** The heatmap analysis showed the expression of most significantly upregulated lncRNAs with the criteria of [log2(FC] ≥ 1 and basal expression ≥ 1 with *Q*-value ≤ 0.05. **(D)** A total of 298 SDE mRNAs and 73 SDE lncRNAs were subjected to the WGCNA. A total of 4 modules were identified by the hierarchical clustering method. **(E)** The histogram showed the gene numbers of each module. **(F)** The heatmap showed the correlation coefficient between module eigenvalues and gastric lesions with *H. pylori* infection. The correlation coefficients and *P*-values are represented in the center of the modules.

To explore the dysregulated lncRNAs involved in the pathogenesis of *H. pylori* infection, we constructed a lncRNA expression profile in AGS cells using *H. pylori* 7.13 or 43504 infection. According to the lncRNA sequencing data, we identified 74 lncRNAs (| log2(FC) | ≥ 1, *Q*-value ≤ 0.001) that were consistently dysregulated in both 7.13- and 43504-infected gastric cells. We showed these SDE lncRNAs between the control and *H. pylori* groups using heatmap analysis (Figure 5A). By analyzing the list of SDE lncRNAs, we found that 53 lncRNAs were consistently upregulated and 20 lncRNAs were consistently downregulated after *H. pylori* 7.13 and 43504 infections (Figure 5B and Supplementary Table S6). To determine the most significantly differentially expressed lncRNA, we set the thresholds as [log2(FC)] ≥ 1, basal expression ≥ 1 with *Q*-value ≤ 0.05. We found that there were 22 differentially expressed lncRNAs after *H. pylori* infection, as shown in Figure 5C and Supplementary Table S7.

### Constructing the lncRNA-mRNA Co-expression Network

It is well reported that lncRNAs could regulated the expression of mRNAs (Wang and Chang, 2011). To explore the potential functions and mechanisms of SDE lncRNAs in *H. pylori*-associated gastric carcinogenesis, we utilized the WGCNA R software package to analysis the expression patterns between these 298 SDE mRNAs (Supplementary Table S2) and 73 lncRNAs (Supplementary Table S5). We revealed five modules following *H. pylori* infection, including Blue, Green, Brown, Turquoise and Yellow (Figure 5D). The blue, brown, green, turquoise, and yellow modules contained 59, 49, 20, 70, and 37 genes, respectively, (Figure 5E and Supplementary Table S8). Module association analysis indicated that these modules seem to be positively correlated with *H. pylori* infection. However, there was no statistical significance (Figure 5F). Furthermore, GO function and KEGG enrichment analysis were conducted to obtain biological function into each module (Supplementary Figure S2). We found that six genes including RELB, CXCL8, TNFAIP3, BIRC3, TRAF1, and NF-KB2 in the turquoise module were significantly enriched in NF-κB signaling pathway (*Q*-value = 0.0003), which is commonly activated to induce acute and chronic gastric inflammation following *H. pylori* infection (Supplementary Figure S2D) (Sokolova and Naumann, 2017). Taken together, these data suggested that all of these modules comprised genes may be involved in the gastric pathogenesis of *H. pylori* infection.

### Expression of the Significantly Differentially Expressed lncRNAs Through *in vitro* Studies

**FIGURE 6:**
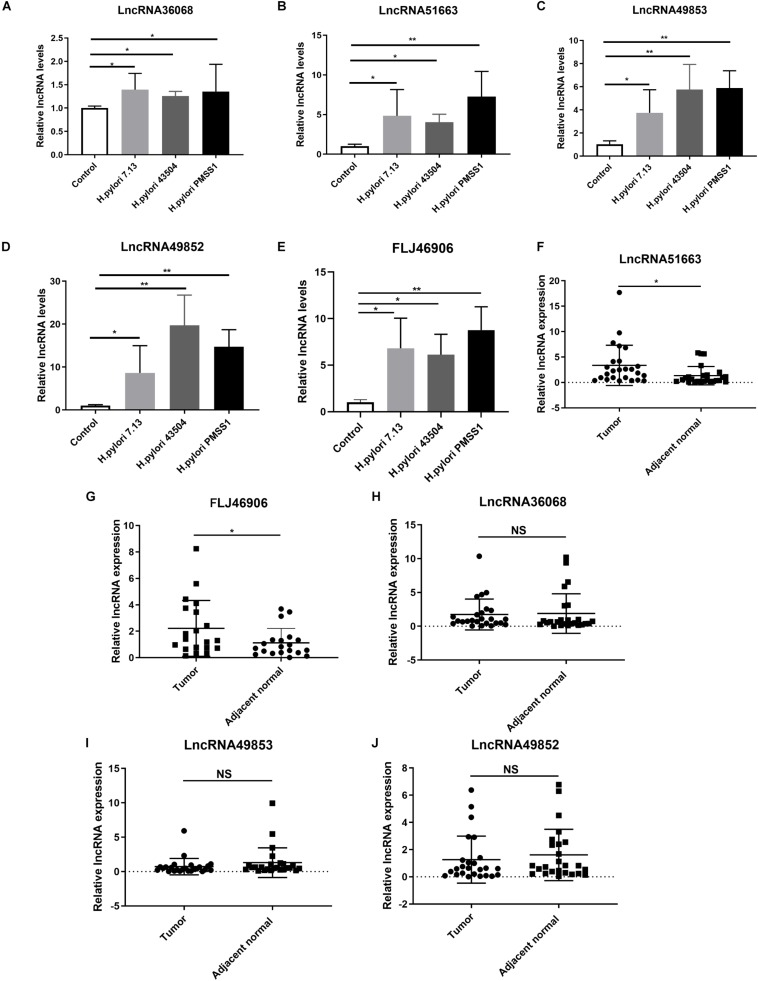
Validation of the significantly differentially expressed lncRNAs. **(A–E)** Gastric epithelial AGS cells were cocultured with the CagA^+^ *H. pylori* 43504, 7.13 and PMSS1 strains for 6 h. The qRT-PCR analysis was performed to assess the expression levels of lncRNA36068 **(A)**, lncRNA51663 **(B)**, lncRNA49853 **(C)**, lncRNA49852 **(D)** and FLJ46906 **(E)**. **(F–J)** The qRT-PCR analysis was performed to determine the expression of lncRNAs, including lncRNA51663 **(F)**, FLJ46906 **(G)**, lncRNA36068 **(H)**, lncRNA49853 **(I)**, lncRNA49852 **(J)**, in 25 human GC samples and matched adjacent non-cancerous tissues. The relative quantification used GAPDH as an internal control. NS, no significance; *p < 0.05; **p < 0.01.

According to the data of SDE lncRNAs in Figure 5C, 5 candidate lncRNAs, including lncRNA36068 (BGIG9606_36068), lncRNA51663 (BGIG9606_51663), lncRNA49853 (BGIG9606_49853), lncRNA49852 (BGIG9606_49852) and FLJ46906, were selected and validated through *in vitro* studies. A qRT-PCR analysis was performed to detect these lncRNAs expression levels in AGS cells following infection with the *H. pylori* 7.13, 43504 or PMSS1 strain. Consistent with the transcriptome sequencing data, we found that *H. pylori* infection significantly increased the expression of lncRNA36068, lncRNA51663, lncRNA49853, lncRNA49852, and FLJ46906 (Figures 6A–E, respectively).

### Expression of the SDE lncRNAs in Human GC Tissues

It is well documented that *H. pylori* infection is the main cause of GC. We next investigated the expression levels of these lncRNAs in human GC tissues. Given that it is difficult to identify these novel lncRNAs from the TCGA database, 25 GC samples and matched adjacent non-cancerous tissues were collected. We also performed qRT-PCR analysis for the expression levels of these lncRNAs. The results showed that the average relative expression of lncRNA51663 and lncRNA FLJ46906 were significantly higher in GC tissues than in adjacent non-cancerous tissues (Figures 6F,G, respectively). However, no significant differences were observed in the expression levels of lncRNA36068, lncRNA49853, and lncRNA49852 between GC and normal tissues (Figures 6H–J, respectively). Taken together, these qRT-PCR analyses validated the results obtained by the RNA-seq data. Given that lncRNA51663 and FLJ46906 were consistently expressed in *H. pylori*-infected cells and GC tissues, they might be involved in gastric carcinogenesis induced by *H. pylori* infection.

## Discussion

It is well established that *H. pylori* infection is associated with a high risk for developing GC. In response to *H. pylori* infection, non-coding RNAs were dysregulated to promote oncogenic cellular processes, which augments the progression to gastric carcinogenesis (Yousefi et al., 2019). lncRNAs, as unique non-coding RNAs, are the key regulators of genes in different physiological and pathological processes. In this study, we performed transcriptome sequencing to identify the expression profiles of lncRNAs and mRNAs in gastric epithelial AGS cells infected with *H. pylori*. To investigate the dysregulated genes involved in the *H. pylori* infection pathogenesis, we identified that 298 SDE mRNAs and 74 lncRNAs were consistently upregulated after infection with *H. pylori* 7.13 and 43504 strains. Furthermore, the most significant differential expression of mRNAs, including DDIT4, NDRG1, CHAC1, IL32, RELB, CTH, and SLC7A1, and lncRNAs including, lncRNA36068, lncRNA51663, lncRNA49853, lncRNA49852, and FLJ46906, were validated by qRT-PCR analyses. We confirmed that *H. pylori* infection induced RELB, SLC7A11 and IL-32 gene levels in cells and INS-GAS mice, which was supported by the results showing a higher expression of these genes in GC tissues. In addition, we found that lncRNA51663 and FLJ46906 were remarkably increased after *H. pylori* infection, which is also consistent with the data of their overexpression in GC tissues compared to adjacent non-cancerous tissues. Together, these results indicated that RELB, SLC7A11, IL-32, lncRNA51663, and lncRNA FLJ46906 might play oncogenic roles in *H. pylori* infection-associated gastric carcinogenesis. Specifically, several other significantly differentially expressed mRNAs (MMP10, LYPD3, ADM2, S100A3 et al.) and lncRNAs (LOC102724908, LOC105371684, et.al.) needed to be verified in the future studies.

Accumulating evidence has shown lncRNAs play crucial roles in multiple diseases. RNA-seq is currently the most widely used tool to analyze the expression profiles of whole-transcriptome mRNAs and lncRNAs (Qian et al., 2019). There are several recent studies investigating the profiles of lncRNAs involved in *H. pylori* infection-associated diseases. Zhu et al. (2015) identified that 303 lncRNAs and 565 mRNAs were aberrantly expressed in GES-1 cells infected with the *H. pylori* 26695 strain. In a preliminary exploration of lncRNAs expression profiles, Zhou et al. (2016) performed a microarray analysis in *H. pylori*-infected and non-infected gastric tissues. The expression of the lncRNA af147447 decreased after *H. pylori* infection, and this lncRNA inhibited GC proliferation and invasion. Moreover, it has been recently reported that the expression profiles of lncRNAs were investigated in *H. pylori* NCTC26695 strain-infected AGS cells. The pro-oncogenic lncRNA THAP9-AS1 was significantly upregulated following *H. pylori* infection and showed an increased expression in GC tissues than in gastritis tissues (Jia et al., 2019). These findings appear to be in disagreement with our RNA-seq results. This discrepancy could be attributed to two factors. First, we used the *H. pylori* strains 7.13 and 43504 in this study for selecting common differential lncRNAs and mRNAs, and these strains were different than those used in other studies. Second, different sequencing platforms and data analyses could play important roles. In this model, we not only explored the expression profiles of lncRNAs and mRNAs associated with *H. pylori* infection, but also validated the most significantly upregulated genes using *in vitro* gastric cells and *in vivo* animal models and human samples.

KEGG enrichment analysis of 298 consistent SDE genes was conducted in this study. We found that several well-known signaling pathways, including the MAPK signaling pathway, PI3K/Akt signaling pathway and NF-κB signaling, were enriched after *H. pylori* infection. However, only cytokine-cytokine receptor interaction and Kaposi’s sarcoma-associated herpesvirus infection were significantly enriched. These results are supported by many previous studies that *H. pylori* infection stimulates to host inflammatory response and induces cytokines expression via the activation of the NF-κB signaling pathway, which contributes to the progression to gastritis and from intestinal metaplasia to hyperplasia (Sakitani et al., 2012; Tafreshi et al., 2018; Yang et al., 2018). Accumulating evidence also indicates that *H. pylori* infection activates the MAPK and PI3K/Akt signaling pathways, further resulting in an aberrant proliferation and cellular apoptosis, thereby triggering gastric carcinogenesis (Yong et al., 2015). This study demonstrates the crucial roles of these pathways in the gastric pathogenesis of *H. pylori* infection.

Moreover, the most significantly differential mRNAs were validated among this study. We found that DDIT4, CTH, IL-32, RELB, and SLC7A11 were upregulated in *H. pylori*-infected cells and INS-GAS mice. There were no significant differences in the levels of DDIT4, CTH, and SLC7A11 in INS-GAS mice. It may be due to the fewer number of animals in this study. Notably, the expression levels of IL-32, RELB, and SLC7A11 were significantly increased in human GC tissues than in normal tissues. These data suggest that the dysregulation of IL-32, RELB, and SLC7A11 might be implicated in gastric carcinogenesis induced by *H. pylori* infection. RELB as a member of NF-κB family, is essential for the constitutive nuclear NF-κB activity (Bren et al., 2001). SLC7A11, as a regulator of glutamine metabolism, could protect cells from oxidative stress by promoting cystine uptake and glutathione biosynthesis (Koppula et al., 2018). IL-32 functions as a novel inflammatory cytokine and is shown to be induced by *H. pylori* infection. Activation of IL-32 could employ the NF-κB signaling pathway to promote gastric inflammation (Sakitani et al., 2012). Taken together, dysregulated RELB, IL-32, and SLC7A11 may mediate inflammatory responses, oxidative stress levels or cellular energy metabolism to potentiate the ability of *H. pylori* to induce gastric carcinoma.

In addition to the mRNA analysis, the most significantly upregulated lncRNAs were verified by qRT-PCR analyses. Due to the failure to design lncRNA primers in mouse species, we used the samples from *H. pylori*-infected cells and human gastric carcinoma patients. We found that lncRNA36068, lncRNA51663, lncRNA49853, lncRNA49852, and FLJ46906 were significantly upregulated after infection with the *H. pylori* 7.13, 43504 or PMSS1 strain. However, only lncRNA51663 and FLJ46906 were consistently increased in human GC tissues than in adjacent normal tissues. Here, our results, for the first time, suggested that the upregulation of lncRNA51663 and FLJ46906 may contribute to *H. pylori*-induced gastric tumorigenesis. Strikingly, the recently identified lncRNA FLJ46906 has been shown to regulate inflammation-related genes, including IL-1β, IL6, CXCL8, TGF-β and ELN, through the mediation of NF-κB and AP-1 in the aging process (Yo and Runger, 2018). We concluded that *H. pylori* infection upregulates the expression of FLJ46906, which activates the NF-κB signaling pathway to induce gastric inflammation and intraepithelial neoplasia. Thus, our study provides insights into the involvement of lncRNAs and mRNAs in the pathogenesis of *H. pylori* infection and has identified several potential biomarkers that might help improve the strategy for the diagnosis and treatment of *H. pylori*-associated gastric neoplasia. Further experiments are needed to be performed to explore the biological functions and specific regulatory mechanisms of these newly discovered mRNAs and lncRNAs in *H. pylori* infection-induced gastric carcinogenesis.

## Data Availability Statement

The raw data supporting the conclusions of this article will be made available by the authors, without undue reservation, to any qualified researcher.

## Ethics Statement

Ethical review and approval was not required for the study on human participants in accordance with the local legislation and institutional requirements. Written informed consent for participation was not required for this study in accordance with the national legislation and the institutional requirements. The animal study was reviewed and approved by the Ethics Committee of The First Affiliated Hospital of Nanchang University.

## Author Contributions

NL conceived and designed the study. NL, YO, and XY performed the qRT-PCR analyses. NL and SC analyzed the RNA-seq data. YO and CH collected the human samples. CP established the INS-GAS mice model infected with *H. pylori*. JH and YZ provided assistance with analyses of the data and technical issues. N-HL supervised and oversaw the study. All the authors critically revised the manuscript and provided intellectual content and read and approved the final manuscript.

## Conflict of Interest

The authors declare that the research was conducted in the absence of any commercial or financial relationships that could be construed as a potential conflict of interest.

## Footnotes

1 https://software.broadinstitute.org/morpheus/

## Supplementary Material

FIGURE S1 **(A)** Identification of RNA types between control and H. pylori-infected groups in AGS cells. **(B)** Pearson’s correlation plot revealing the correlation (*r*) values between samples. **(C)** PCA analysis for the RNA-seq data that characterizes the trends exhibited by genes expression profiles of control, *H. pylori* 43504 infection and *H. pylori* 7.13 infection groups. **(D)** The heatmap shows the expression levels of significantly differentially expressed genes.

FIGURE S2 GO function and KEGG enrichment analysis for genes in the object module. **(A)** Blue module; **(B)** Brown module; **(C)** Green module; **(D)** Turquoise module; **(E)** Yellow module. The *x*-axis shows the rich ratio of gene numbers annotated in a pathway term. The *y*-axis shows the GO function and KEGG pathway terms. The size and color of the bubbles represent the gene numbers enriched in the pathway and enrichment significance, respectively.

TABLE S1 The PCR primers for SDE mRNAs and lncRNAs validation.

TABLE S2 The significantly differentially expressed mRNAs list following *H. pylori* 7.13 and 43504 infection.

TABLE S3 The significantly differentially expressed mRNAs list between uninfected and *H. pylori* 7.13-infected gastric cells.

TABLE S4 The significantly differentially expressed mRNAs list between uninfected and *H. pylori* 43504-infected gastric cells.

TABLE S5 The most significantly differential expressed mRNAs following *H. pylori* 7.13 and 43504 infection.

TABLE S6 The significantly differentially expressed lncRNAs list following *H. pylori* 7.13 and 43504 infection.

TABLE S7 The most significantly differential expressed lncRNAs following *H. pylori* 7.13 and 43504 infection.

TABLE S8 The module eigengenes of modules using WGCNA analysis.
