# Supplementary figures and images for: Integrative Analysis of Differential lncRNA/mRNA Expression Profiling in Helicobacter pylori Infection-Associated Gastric Carcinogenesis
Source: Front Microbiol. 2020 May 8;11:880. doi: 10.3389/fmicb.2020.00880 (PMC7225608; doi:10.3389/fmicb.2020.00880)

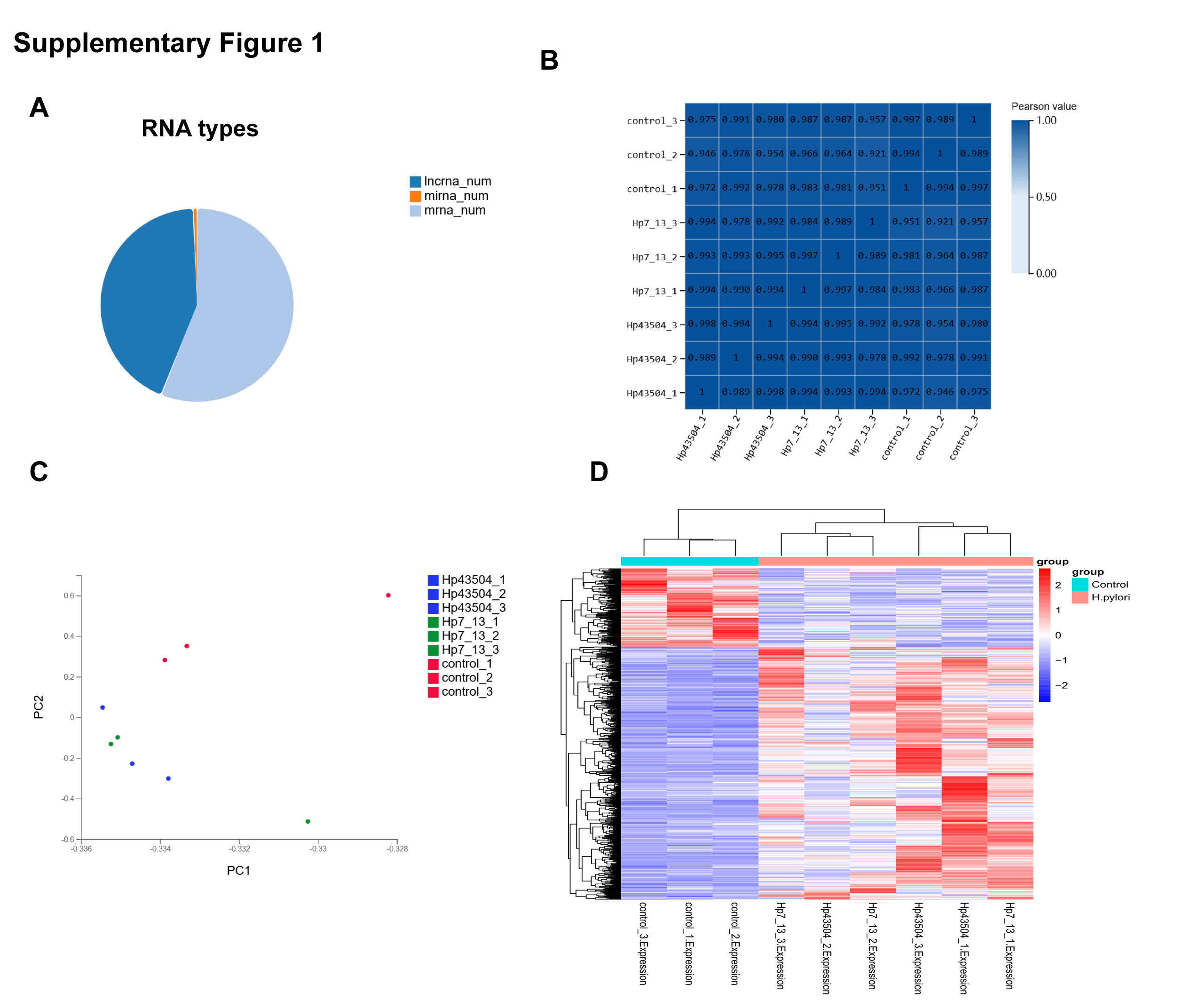

Supplement: FIGURE S1 — (A) Identification of RNA types between control and H. pylori-infected groups in AGS cells. (B) Pearson’s correlation plot revealing the correlation (r) values between samples. (C) PCA analysis for the RNA-seq data that characterizes the trends exhibited by genes expression profiles of control, H. pylori 43504 infection and H. pylori 7.13 infection groups. (D) The heatmap shows the expression levels of significantly differentially expressed genes. [file Image_1.TIF]

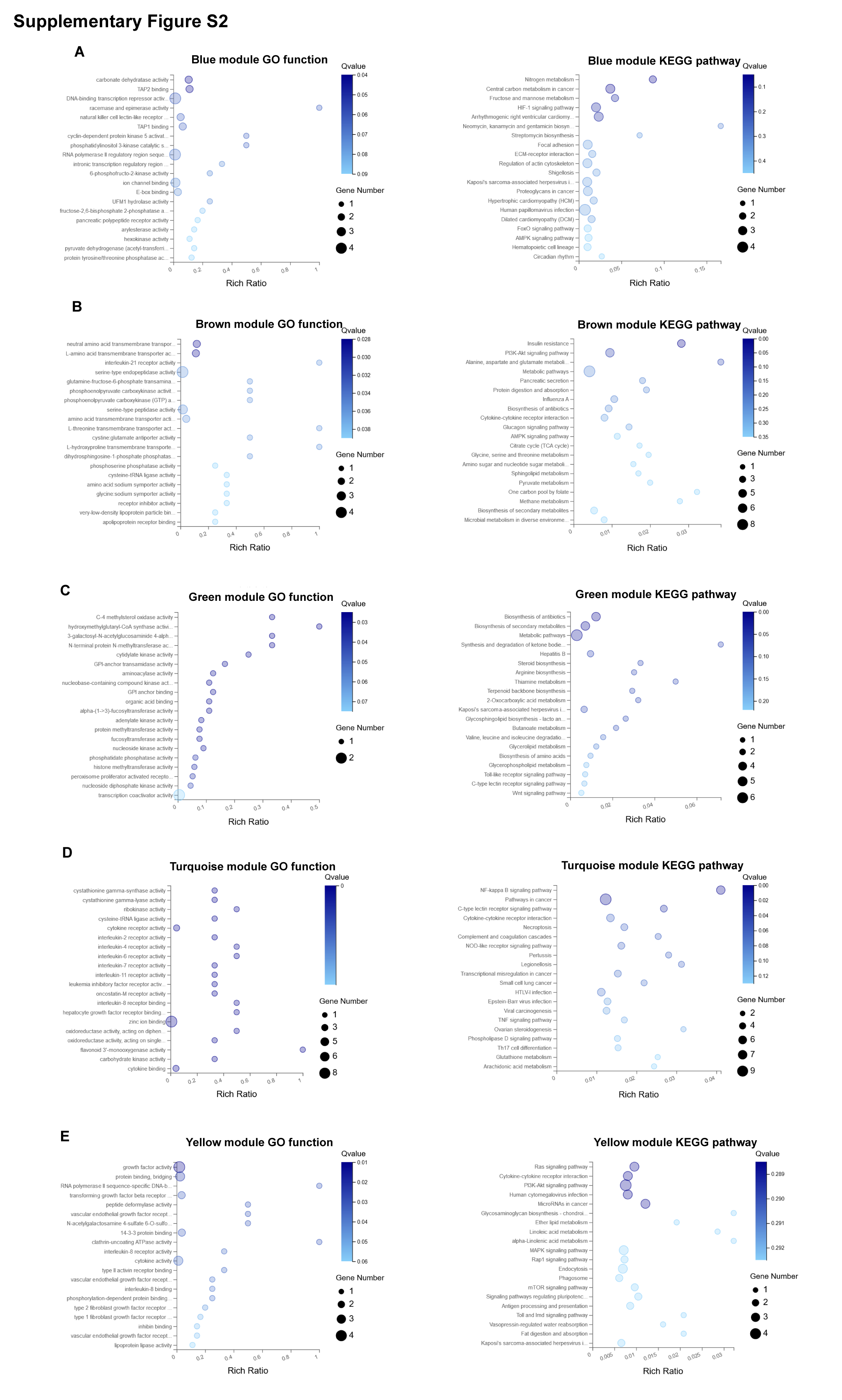

Supplement: FIGURE S2 — GO function and KEGG enrichment analysis for genes in the object module. (A) Blue module; (B) Brown module; (C) Green module; (D) Turquoise module; (E) Yellow module. The x-axis shows the rich ratio of gene numbers annotated in a pathway term. The y-axis shows the GO function and KEGG pathway terms. The size and color of the bubbles represent the gene numbers enriched in the pathway and enrichment significance, respectively. [file Image_2.TIF]
